# Supplementary material for: Comparative genomics provides new insights into the diversity, physiology, and sexuality of the only industrially exploited tremellomycete: Phaffia rhodozyma
Source: BMC Genomics. 2016 Nov 9;17:901. doi: 10.1186/s12864-016-3244-7 (PMC5103461; doi:10.1186/s12864-016-3244-7)
Supplement: Additional file 6: — List of orphan genes with links to PFAM (related to Additional file 1: Table S1). (ZIP 1428 kb) [file 12864_2016_3244_MOESM6_ESM.zip › PFAM_UNIQUE/G05668_P.html]

#                                                                            --- full sequence --- -------------- this domain -------------   hmm coord   ali coord   env coord
  
# target name        accession   tlen query name           accession   qlen   E-value  score  bias   #  of  c-Evalue  i-Evalue  score  bias  from    to  from    to  from    to  acc description of target
  
#------------------- ---------- ----- -------------------- ---------- ----- --------- ------ ----- --- --- --------- --------- ------ ----- ----- ----- ----- ----- ----- ----- ---- ---------------------
  
G05668\_P             -            157 HTH\_32               PF13565.1     77    0.0069   15.8   0.1   1   2   1.2e-06    0.0069   15.8   0.1    32    77    26    67     4    67 0.80 - Homeodomain-like domain  
G05668\_P             -            157 HTH\_32               PF13565.1     77    0.0069   15.8   0.1   2   2      0.43   2.5e+03   -2.0   0.7     6    20   125   144   123   155 0.42 - Homeodomain-like domain  
G05668\_P             -            157 HTH\_40               PF14493.1     91   0.00071   18.4   0.0   1   1   1.7e-07     0.001   18.0   0.0     3    52    31    83    29   118 0.82 - Helix-turn-helix domain  
G05668\_P             -            157 UPF0175              PF03683.8     76    0.0051   14.9   0.1   1   1     2e-06     0.012   13.8   0.1     9    36    18    44    10    48 0.78 - Uncharacterised protein family (UPF0175)
